# Intermediate CAG Repeat Expansion in the *ATXN2* Gene Is a Unique Genetic Risk Factor for ALS−A Systematic Review and Meta-Analysis of Observational Studies

**DOI:** 10.1371/journal.pone.0105534

**Published:** 2014-08-22

**Authors:** Ming-Dong Wang, James Gomes, Neil R. Cashman, Julian Little, Daniel Krewski

**Affiliations:** 1 Department of Epidemiology and Community Medicine, Faculty of Medicine, University of Ottawa, Ottawa, Ontario, Canada; 2 Department of Medicine, University of British Columbia, Vancouver, Canada; UMCG, Netherlands

## Abstract

Amyotrophic lateral sclerosis (ALS) is a rare degenerative condition of the motor neurons. Over 10% of ALS cases are linked to monogenic mutations, with the remainder thought to be due to other risk factors, including environmental factors, genetic polymorphisms, and possibly gene-environmental interactions. We examined the association between ALS and an intermediate CAG repeat expansion in the *ATXN2* gene using a meta-analytic approach. Observational studies were searched with relevant disease and gene terms from MEDLINE, EMBASE, and PsycINFO from January 2010 through to January 2014. All identified articles were screened using disease terms, gene terms, population information, and CAG repeat information according to PRISMA guidelines. The final list of 17 articles was further evaluated based on the study location, time period, and authors to exclude multiple usage of the same study populations: 13 relevant articles were retained for this study. The range 30–33 CAG repeats in the *ATXN2* gene was most strongly associated with ALS. The meta-analysis revealed that the presence of an intermediate CAG repeat (30-33) in the *ATXN2* gene was associated with an increased risk of ALS [odds ratio (OR) = 4.44, 95%CI: 2.91–6.76)] in Caucasian ALS patients. There was no significant difference in the association of this CAG intermediate repeat expansion in the *ATXN2* gene between familial ALS cases (OR = 3.59, 1.58–8.17) and sporadic ALS cases (OR = 3.16, 1.88–5.32). These results indicate that the presence of intermediate CAG repeat expansion in the *ATXN2* gene is a specific genetic risk factor for ALS, unlike monogenic mutations with an autosomal dominant transmission mode, which cause a more severe phenotype of ALS, with a higher prevalence in familial ALS.

## Introduction

Amyotrophic lateral sclerosis (ALS) is a rare neurological degenerative condition of the motor neurons leading to paralysis of skeletal muscles, characterized by rapid irreversible progression in most cases [1]. Although the causes of ALS are largely unknown, it is estimated that genetic and environmental risk factors contribute roughly equally to the development of ALS [2], [3]. Over the last 20 years, over 10% of all ALS cases [∼68% of familial ALS (fALS), ∼11% of sporadic ALS (sALS)] have been linked to monogenic mutations in one of 30 or so genes [4]–[7]. The mutation frequencies for most known causal genes for ALS are rare among ALS cases, with most accounting for less than 1% of ALS cases. Relatively common mutations occur in the *SOD1* (superoxide dismutase 1) gene, which represents the first genetic factor linked to ALS in 1993, and for which there is a valid animal model [8]. Nearly 200 mutations in this gene have been reported in literature [9], [10]. The mutations in this gene are responsible for about 2–5% of all ALS cases across all races [11], [12], *SOD1* remained the most common mutated gene related to ALS until the discovery of the GGGGCC hexanucleotide repeat expansion in *C9orf72* in ALS patients in 2011 [13], [14]. We now know that the most common monogenic mutated gene in Caucasians with ALS is the GGGGCC hexanucleotide repeat expansion in the *C9orf72* [13], [14], which is responsible for over 30% of fALS cases, and about 6.0% of sALS in Caucasian populations [7], [15], [16]. This repeat mutation arose in Northern Europe a few thousand years ago [17], and thus occurs with a much lower frequency in Asian ALS populations [18]–[20], in particular, the mutation frequency has been shown to be extremely low in Chinese, Indian, Japanese and Korean ALS patients [16], [19]–[23]. This mutation is also associated with about 25% of familial frontotemporal lobe dementia (FTLD) and about 6% sporadic FTLD [16], [24]. Among 297 carriers of the hexanucleotide repeat expansion in *C9orf72*, the diagnoses of ALS, ALS/FTLD and FTLD were 43%, 26% and 31%, respectively [25]. Consequently, the incidence of this mutation in Caucasians with ALS may have been underestimated, since the clinical determination of ALS was mainly based on differential diagnosis. Other relatively common mutations occurred in the *TARDBP* (5% of fALS, 2% of sALS), and *FUS* (1–5% of fALS, 1.0% of sALS) genes [6], [7]. Both gene products belong to the RNA/DNA binding protein family, and are involved in the pathological protein aggregation pathway in ALS patients [26]. The contribution of other types of genetic risk factors (polygenic gene mutations, variants of polymorphic genes) to the burden of ALS has also been investigated in over 130 studies involving about 400 variants in more than 100 genes [9], [27], [28]. The abnormal expression of this type of gene variant alone is likely to be insufficient to initiate the onset of ALS; rather, exposure to certain environmental agents is thought to render genetic variant carriers susceptible to ALS [29] . To date, extensive studies have not revealed a definitive, universally accepted environmental risk factor for ALS, although many environmental risk factors, such as exposure to heavy metals, exposure to pesticides, exposure to solvent, intake of biological toxicants, history of head injury, smoking, and military service have been reported to be associated with ALS in some studies [30]–[35].

Variants of approximately 30 genes have been found to be associated with ALS development in at least one study. These associations have been complied in a website summarizing known genetic risk factors for ALS [36] and have been documented in many peer-reviewed publications. These associations need to be verified in further epidemiological studies, even for the most credible gene variants for sporadic ALS, such as the Paraoxonase 1 polymorphism [37], the H63D polymorphism in *HFE* [38], [39], and variants of the gene encoding vascular endothelial growth factor [40].

Over the last few years, repeat expansions in two genes in ALS patients have been investigated intensively. One is the GGGGCC hexanucleotide repeat expansion in the upstream of the *C9orf72* coding region [13], [41], which causes ALS among Caucasians and populations with a Spanish ethnic background [42], but is rare among ALS cases with other ethnic backgrounds [19]–[21]. The mutation frequencies of this gene among ALS patients with various ethnic backgrounds have been summarized [15], [16]. The second repeat mutated gene demonstrates a higher occurrence of intermediate CAG (coding for glutamine) repeats (polyglutamine, polyQ) in the 5 prime terminal of the *ATXN2* gene in ALS patients [43]. However, there is currently no agreement about which range of the intermediate CAG repeat is associated with ALS, nor its relative prevalence in fALS and sALS cases. To address these issues, we examined the association between this intermediate CAG repeat expansion in the *ATXN2* gene and ALS using meta-analysis.

## Materials and Methods

This systematic review and meta-analysis was conducted following the PRISMA guideline [44], major features of which are listed in Supporting Information (Checklist S1). Articles related to the association between ALS (disease terms: ALS, MND, amyotrophic lateral sclerosis, motor neuron disease, motoneurone disease, or Lou Gehrig's disease) and intermediate CAG repeat expansion in the *ATXN2* gene (gene terms: ataxin-2, *ATXN2*, *ATX2*, *SCA2, ASL13, TNRC13*, polyglutamine, polyQ, CAG repeat) were searched in MEDLINE, EMBASE, PsycINFO, Pubmed, Hugenet and Google Scholar from January 1, 2010 through to January 20, 2014. The detailed search strategies are described in Supporting Information (Search S1). The retrieved articles were exported to Refworks for screening. First, duplicates were removed by comparing the authors and titles of adjacent records after sorting the articles by first author. Second, irrelevant articles were excluded by reading the titles and abstracts of records against our inclusion/exclusion criteria (at least one disease term and one gene term were required for inclusion). Third, relevant or ambiguous articles were retained for next level screening by examining the full PDF copy of the article. At this level of screening, letters to editors, commentaries, generic reviews, case reports only, conference abstracts, mechanistic and animal studies were excluded by examining full PDF. Articles without one of the essential characteristics of the study population (study location, case recruitment time, and method of recruitment) were also excluded at this screening level. Fourth, the remaining articles were assessed by examining the authors, research groups, country of study, case ascertainment, population recruitment periods, analytic methods, results, and conclusions based on the previous publication [45]. Since ALS is a rare neurological condition, care was taken to avoid inclusion of multiple articles with same study participants. When two or more papers were from the same group of authors, special attention was given to information on the centers involved in, and periods of recruitment of the study participants. Any article with duplicate uses of ALS patient populations or any ambiguous description about the studied population was excluded. Fifth, data from the final list of included articles were extracted (including first author, published year, country, CAG repeat information, case and control information, and main results) (Table S1). The screening and data extraction steps were assessed by second reviewer, based on a random sample of 4 of 33 articles available at these steps. Finally, data were synthesized using meta-analytic approaches based on both random and fixed effects models [44]. The synthetic odds ratio (OR), Tau^2^, I^2^ and χ^2^ statistics were reported, and potential bias due to small study effects was examined using funnel plots. Comparisons among population subgroups defined by geographic region and types of ALS were also made.

## Results

### 1. Studies selected for meta-analysis

**Figure 1:**
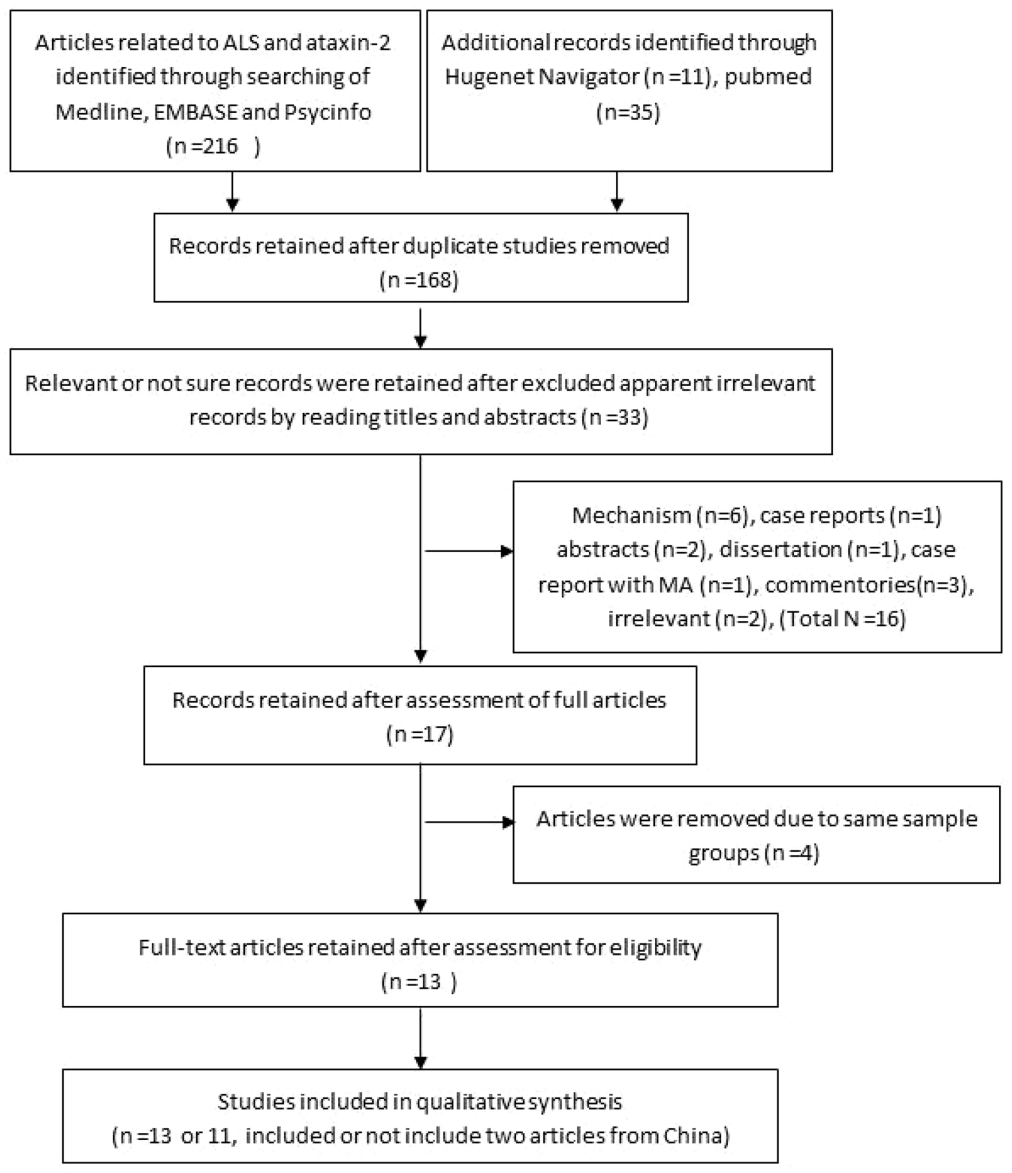
Flow chart for ataxin-2 and ALS related article search, screen, evaluation, and data analysis.

A flow diagram summarizing article search, screen, selection, assessment, data extraction and analysis is given in Figure 1. No previous systematic review was identified, except one meta-analysis [46]. Seventeen relevant articles were selected for inclusion in the present study (Table S1). Three studies were excluded because of multiple uses of same populations. One study was designed to test the presence of intermediate CAG in ALS-FTLD and familial ALS only, and was also excluded from the meta-analysis. The data from remaining 13 articles (first author, published year, country, CAG repeat information, case and control information, main results), all of which followed a case-control design, were extracted and synthesized by meta-analysis.

### 2. Determination of the range of intermediate CAG repeats in the *ATXN2* gene that is associated with ALS

Since Elden and colleagues reported that a 27–33 CAG repeat expansion in the *ATXN2* gene was associated with sporadic ALS in 2010 [43], [47], about two dozen studies have sought to verify this association [46], [48]–[60], investigate the mechanisms by which ALS may develop [61], [62], or investigate different clinical manifestations in ALS patients carrying CAG repeat expansions in the *ATXN2* gene [61]–[64]. The initial study focused on the range 27–33 in the number of CAG repeats, and determined this range as an intermediate CAG (PolyQ) repeat using ROC curves. However, no consensus has been reached regarding which CAG repeat range is associated with ALS [43], [48], [52], [65].

**Figure 2:**
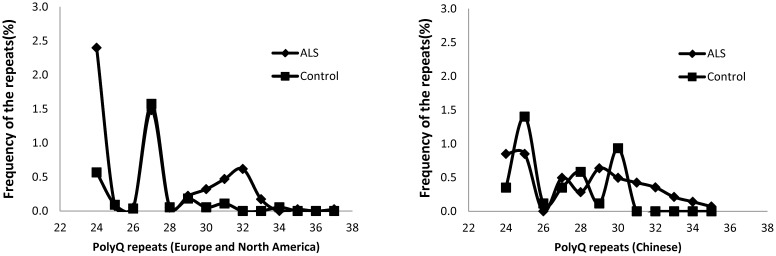
Identification of the differences of the presence of intermediate CAG repeats and potential ethnic variances in the *ATXN2* gene among ALS and control subjects. The data about the number of CAG repeats in ALS and control subjects from 9 included studies in Caucacians and from 2 included studies from Chinese were summarized and plotted separately (left panel; right panel).

To address this issue, we compared the CAG repeat range between ALS patients and controls based on the information from the 13 studies selected for inclusion in Figure 2 [46], [48]–[60]. A high frequency of 27Q repeats was noticed in ALS patients, Parkinson's disease (PD) patients, and controls in Europe and North America (Figure 2a), but not in Chinese ALS patients or healthy controls (Figure 2b), where the peak of 27 CAG repeats in the *ATXN*2 gene was essentially absent [58], [59]. These results indicated that the peak of 27 CAG repeats is not an ALS disease-specific CAG repeat, but might be a specific genomic marker of ethnicity [66]. Although outside the scope of the present study, the higher rate of 24 or 25 CAG repeats in ALS patients (Figure 2a, 2b) may be relevant to the transmission of new larger CAG repeats to the next generation. Based on the 9 studies in North America and Europe in which detailed repeat information could be identified (Table S1), the pattern of CAG 30–33 repeats in the *ATXN2* gene in ALS patients appeared to be different than in controls (Figure 2a). These data suggest that 30–33 CAG repeats is associated with ALS among Caucasian populations. Ethnic variation appears to exist, with the major CAG repeat range associated with ALS appearing to be 31–36 in Chinese subjects (Figure 2b) [58], [59]. Meta-analyses were then conducted, with and without these two studies from China, to explore this association in quantitative terms.

### 3. The synthesized odds ratio (OR) estimate from multiple studies based on meta-analysis

**Figure 3:**
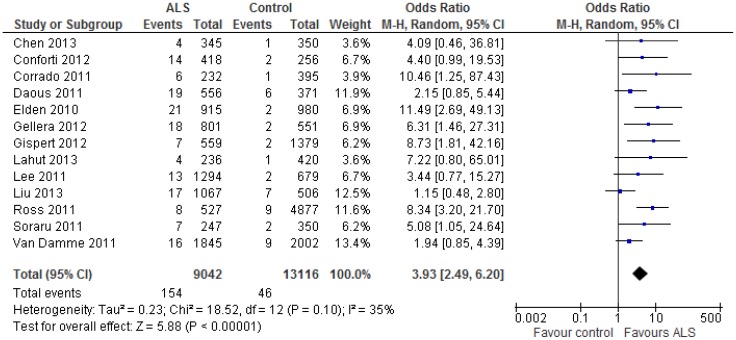
The presence of intermediate CAG 30–33 repeats in the *ATXN2* gene is associated with ALS. The data of intermediate CAG 30–33 repeats in *ATXN2* were extracted from 13 included studies and the OR of intermediate CAG repeat among ALS and control subjects was synthesized with meta-analysis using random effects model. Similar results were also obtained when use fixed effects model (data not shown).

The 13 studies included in the present meta-analysis involved a total of 154 positive carriers of 30–33 CAG repeats in the *ATXN2* gene among 9,042 ALS cases (including fALS cases with no known monogenic mutations), and 46 positive carriers of the same CAG repeats among 13,116 controls. The crude prevalence rate of the CAG repeats (1.70%) in ALS cases was significantly higher than in controls (0.35%, P<0.0001). Comparing cases to controls, the odds ratio (OR) for ALS in relation to the 30–33 CAG repeat sequence was estimated to be OR = 3.93 (95% CI: 2.49–6.20) using a random effects model (Figure 3), with moderate heterogeneity across included studies as reflected by the I^2^ statistic [35%, P = 0.10]. Similar results (OR = 3.60, 2.54–5.09) were obtained with a fixed effects model (data not shown).

**Figure 4:**
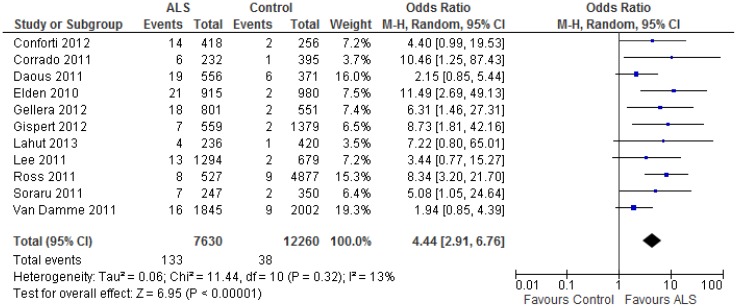
The heterogeneity among included studies was reduced dramatically after excluding two studies from China. This meta-analysis was conducted using same protocol as in Figure 3 except excluding two studies with patients with Chinese background. Please refer to Figure 3.

When the two studies from China were excluded, heterogeneity decreased (I^2^ = 13%). The remaining 11 articles reported a total of 134 positive carriers of 30–33 CAG repeats among 7,625 ALS cases (including sALS and fALS cases), and 37 positive carriers of the same CAG repeats among 12,555 controls (see Table S1). Excluding these studies, the odds ratio was estimated to be 4.44 (2.91–6.76) using a random effects model (Figure 4). A similar estimate was obtained with a fixed effects model (data not shown). Funnel plots failed to identify evidence of small study effects (data not shown).

### 4. The prevalence of intermediate CAG repeat in the *ATXN2* gene in fALS cases is not different from that in sALS cases

**Figure 5:**
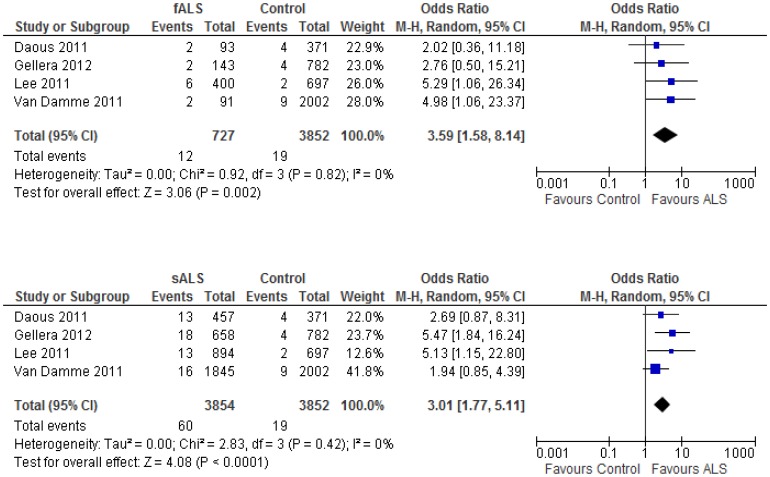
The synthesized OR of the presence of intermediate CAG repeats with meta-analysis is not different from FALS and SALS cases in the *ATXN2* gene. The relative risks (OR) among fALS cases (top panel) or sALS cases (lower Panel) compared to controls were synthesized with meta-analysis using extracted data from 4 included case-control studies. The results from random effects model were presented. Similar results were also obtained when use fixed effects model (data not shown).

Based on the above analyses, we concluded that the intermediate 30–33 CAG repeat expansion in the *ATXN2* gene is a genetic risk factor for ALS. Further analyses were undertaken in an attempt to compare differences in risk between fALS and sALS. High CAG repeat expansions (usually greater than 34) in coding regions of the *ATXN2* gene are the cause of spinocerebellar ataxia type 2 (SCA2) [67], transmitted in an autosomal dominant manner. However, if intermediate CAG 30–33 repeat expansion of *ATXN2* also causes ALS in an autosomal dominant mode, then it might be expected that its prevalence in fALS cases would be higher than that in sALS cases (For example, the frequency of *SOD1* mutations is higher in fALS than in sALS patients). To address this issue, we identified four included articles that provided relevant genomic information for fALS (defined as two or more ALS cases identified in a family) and sALS cases. Meta-analysis using a random effects model showed that the OR for the presence of the intermediate CAG repeat expansion in fALS cases [OR = 3.59 (1.58–8.14)] was not significantly higher than the OR in sALS cases [OR = 3.01 (1.77–5.11)] (Figure 5). The pooled prevalence of the intermediate CAG repeats was 1.32% and 1.58% among sALS and fALS cases, respectively, which are not significantly different from each other (χ^2^ = 0.25, p = 0.62). There was no evidence of heterogeneity (I^2^ = 0%) or publication bias across the four included studies. Similar results were also obtained with a fixed effects model (data not shown).

### 5. The association between CAG repeat length and the age at onset or survival time among ALS patients remains unclear

Out of 13 included articles, 9 articles provided partial numerical data about the age of onset of ALS (116 ALS cases), disease duration (29 cases), and the corresponding CAG repeat numbers in *ATXN2* (27,31,35,36,38,40,43–45) (Table S2). Although linear regression analysis showed that the number of CAG repeats in the *ATXN2* gene was associated with neither the age at onset (R^2^ = 0.004) nor the disease duration (R^2^ = 0.02), these data are insufficient in our opinion to draw firm conclusions about these associations (see Table S2 for further details). No differences in disease progression between ALS patients with and without the intermediate CAG repeat expansion in *ATXN2* were noted.

## Discussion and Conclusions

In this study, we synthesized data from published articles related to intermediate CAG repeat expansions in the *ATXN2* gene in individuals with ALS, and found that an intermediate CAG expansion with a range of 30–33 repeats was associated with an increased risk of ALS. Since significant differences with respect to the prevalence of these expansions among fALS cases compared to sALS cases were not identified, heritability is unlike the dominant autosomal transmission mode of inheritance of SCA2 observed for CAG expansions greater than 34 repeats in the *ATXN2* gene. The intermediate CAG repeat may have been inherited differently in relation to ALS (see discussion below). Therefore, the intermediate CAG repeat expansion in the *ATXN2* gene may not be a simple modulator of ALS [65], [68]. We conclude that the intermediate CAG repeat expansion in *ATXN2* is a unique genetic risk factor for ALS.

High CAG repeat expansions (usually greater than 34 CAG repeat) in the *ATXN2* gene [67], transmitted in an autosomal dominant manner, is the cause of spinocerebellar ataxia type 2 (SCA2), a progressive neurodegenerative disease of the cerebellum, brain stem and spinal cord, with a somewhat higher incidence in Cuba than elsewhere [46], [60], [69]. The intermediate expansion of CAG repeats in SCA2 cases has also been reported [70], [71], and coincidence of ALS and SCA2 patients in the same family has been observed [72], indicating a certain degree of overlap between SCA2 and ALS with CAG repeat expansion in the *ATXN2* gene. The expansion of CAG repeat in the *ATXN2* gene leads to the production of elongated polyglutamine (polyQ) in the corresponding protein. The locus of the *ATXN2* gene has been mapped to chromosome 12, but the function of the *ATXN2* genes product is not known [73]. The CAG repeats in the *ATXN2* gene are variable in size. The length of the allele in normal subjects was found to range from 14 to 31 repeats, with over 90% of normal subjects demonstrating alleles with 22 repeats [67]. The SCA2 disease allele usually increases its size when transmitted to successive generations, and the longer expansions associate with earlier onset and more severe SCA2 disease in subsequent generations [74], different from the ALS cases with intermediate CAG repeat expansion (see Result section 5). Whether or not the intermediate repeat length of CAG (within the normal range, up to 31, and ≥33 for SCA2) was associated with some disease conditions was unknown until Elden and colleagues [43] first linked the intermediate CAG repeat expansion (27–33) to ALS in 2010. Other follow-up reports generally confirmed this initial finding, but there is no consensus with regards to which repeat length is associated with ALS. One study using ROC (receiver operating characteristic) curves showed that more than 29 CAG repeats in the *ATXN2* gene was associated with an increased risk of ALS [54]; however, other studies have linked ALS with greater than 30 CAG repeats in Italy [52], 30–35 CAG repeats in Germany [65], and ≥28 CAG repeats in Italy [48]. Here, we used 30–33 repeats as the cut-off for association with ALS because the difference between ALS cases and corresponding controls was not apparent when the CAG repeat in *ATXN2* is less than 29 or greater than 34. Nonetheless, this intermediate CAG range for ALS might vary in different studies due to ethnicity [58], [59].

The products of the *ATXN2* gene with intermediate CAG repeat expansion may not act like a typical gene modulator [65]. The first study conducted by Elden and colleagues suggested that intermediate CAG repeats were a modulator for ALS [43]. Further experiments showed such repeats might regulate RNA processing [65] and intracellular vesicular trafficking [64]. Importantly, *in vitro* experiments showed that the expression products of *ATXN2* with intermediate CAG repeats could interact with *FUS* [75], a DNA/RNA binding protein, which when mutated could cause fALS as a result of impaired RNA intracellular trafficking [75], [76]. In addition, *TDP-43* cytoplasmic inclusions in motor neurons of ALS patients harboring intermediate PolyQ repeats primarily showed skein-like or filamentous *TDP-43* pathology, whereas ALS cases without ataxin-2 polyQ expansions (n = 13) exhibited abundant large round *TDP-43* inclusions [63], [77], and accumulated activated caspase 3 in motor neurons, an upstream event in the *TDP-43* disease pathway. The product of *ATXN2* with intermediate CAG repeat expansion could increase the presence of *TDP-43* in the cytoplasm in a RNA dependent manner [43]. Moreover, plasma TDP-43 protein was robustly associated with CAG repeat length [43]. These biochemical observations indicated that the expression products of *ATXN2* with intermediate CAG expansions interact with two important genes (*TDP-43* and *FUS*) in ALS pathogenesis. However, it seems that ataxin-2 with intermediate polyQ does not function like a typical modulator of other genetic risk factors for ALS (specifically *TDP-43* and *FUS*), because the mutation of a modulator alone should not cause primary outcome of interest (i.e., ALS). Even if the expression products of *ATXN2* with intermediate CAG expansion were a modulator of *TDP-43* or *FUS*, it must be a weak or very specific modulator, because differences in clinical features of ALS, such as age at onset or survival time, between the CAG repeat carriers and non-carriers have not been established (see Result section 5). During the course of finalizing the present paper, a new article describing the coexistence of intermediate CAG repeat expansion in the *ATXN2* gene and the hexanucleotide repeat expansion in *C9orf72* was published online [25]. Compared to controls, the frequency (∼2%) of intermediate CAG repeat expansions in the *ATXN2* gene was higher among the carriers of the GGGGCC hexanucleotide repeat expansion in *C9orf72* [25]. However, this study did not provide clear evidence that the intermediate CAG repeat expansion in the *ATXN2* gene was a modifier to the disease caused by the hexanucleotide repeat expansion in gene *C9orf72*.

If the *ATXN2* gene with intermediate CAG repeat expansion associated with ALS were transmitted in an autosomal dominant manner, as in the case of *SOD1* mutations, or as in the case of SCA2 for longer CAG repeat expansions in SCA2, then we would expect that the prevalence of the intermediate CAG repeat expansion in the *ATXN2* gene be higher in fALS cases than in sALS cases, as is the case for other genetic risk factors for ALS, such as mutations in *SOD1* and *C9orf72*. Although studies from Cuba and the United States suggested a dominant mode of inheritance [43], [69], [72], our meta-analyses did not show a higher prevalence of the CAG intermediate repeat expansion in fALS cases, as compared to sALS cases. Therefore, the intermediate CAG repeat expansion in the *ATXN2* gene may not be a typical independent classic genetic risk factor transmitted in an autosomal dominant manner. Further studies with well diagnosed fALS cases are required to determine the mode of transmission of the intermediate CAG repeats in the *ATXN2* gene.

The intermediate repeats in *ATXN2* may be transmitted in recessive mode. Van Damme [55] described a consanguineous married couple, the father suffering from an ALS-like disease with two affected sons [elder brother (79 years old) with CAG repeat alleles 31∶33, onset at 75 years, pedigree (middle son, 77 years old) with CAG repeat alleles 33∶33, onset at 71 years] and a normal carrier younger brother (75 years) with 22∶33 CAG repeat alleles. These observations suggest a recessive transmission mode, unlike a SCA2 family with longer CAG repeats descried in the same article [55]. The transmission of intermediate CAG repeat expansion of *ATXN2* gene might behave like the *Asp90Ala* mutation in *SOD1*, which was transmitted in a recessive manner in the North Scandinavian population, but in a dominant manner in other regions [78]. The longer CAG repeat expansions for SCA2 are transmitted in a dominant manner, whereas this is not the case for ALS with intermediate CAG repeat in *ATXN2*. However, we could not rule out the possibility of lower inheritance penetration in a dominant autosomal transmission mode.

The different modes of transmission might be dictated by the different nature of the CAG repeat expansions in ALS and SCA2. Compared to the longer CAG repeats in SCA2 patients, the unique feature in ALS patients with intermediate CAG repeats in the *ATXN2* gene is that the intermediate CAG repeats in ALS patients were interrupted by a CAA triplet in almost all ALS cases with this CAG intermediate repeat expansion [62]. This finding has been confirmed by other similar observations [47]. The *ATXN2* gene without CAA triplets, which stabilizes the CAG repeats [79], may produce ataxin-2 protein with longer PolyQ repeats which is more toxic to the host cells, compared to the ataxin-2 protein produced by the gene with the CAA triplet. This type of triplet is less common in longer CAG repeats in the *ATXN2* gene in SCA2 patients [46], [69], [80], [81]. The CAA interruption may explain why the intermediate CAG repeat expansion in *ATXN2* is associated with ALS, and does not generally produce a mild type of SCA2. Other mechanisms may also influence this comparison. For example, the co-occurrence of fALS with SCA2, or with Parkinson disease, would lead to non-diagnosis of ALS in fALS [72], [82], [83], thus underestimating the occurrence of the intermediate CAG repeats among familial ALS cases.

Collectively, the results of the present study suggest that CAG intermediate (30–33) repeat expansions in the *ATXN2* gene is a genetic risk factor for ALS. During the course of this work, a meta-analysis of the association between intermediate CAG repeats and ALS for a family with 3 ALS cases linked to CAG repeat mutations in *ATXN2*, apparently with an autosomal dominant transmission mode, was reported from Cuba [46]. This article adopted a similar inclusion and exclusion strategy, and identified many of the same studies as were used in the present meta-analysis, with the exception that we also included the two most recent published studies [48], [58]. However, that study compared the synthetic ORs to determine the cut-off for the intermediate CAG repeat expansion (≥30 CAG repeats) that associates with ALS, compared the range used in the present analysis (30–33 CAG repeats). Because they also included gene carriers with more than 34 CAG repeats (which usually causes SCA2), the synthetic risk was artificially underestimated (OR = 2.16), as compared to our estimate (OR = 4.44, Figure 4). We argue that our strategy of determining the optimal range of the intermediate CAG repeats for ALS is preferable, because it is known that if the number of CAG repeats is greater than 34, then the repeat is more likely to be associated with SCA2, rather than ALS [52].

Further studies are needed to verify our conclusion that intermediate CAG repeat expansion in the *ATXN2* gene is a unique genetic risk factor for ALS. Future research should focus on the mechanisms involved in the etiology of ALS among intermediate CAG repeat carriers, and explore the variation in repeats among ALS patients with different ethnic backgrounds. Since our conclusions were drawn mainly from studies conducted in Europe, North America, and China, further observational studies from other regions would be helpful in establishing the generalizability of our findings.

## Supporting Information

Table S1 **Summary of studies included in meta-analyses of the ATXN2 gene as a risk factor for ALS.**
(DOCX)

Table S2 **Age at onset, disease duration and CAG repeat number in ALS patients.**
(DOCX)

Checklist S1 **PRISMA checklist and the section location of each item in the manuscript.**
(DOCX)

Search S1 **Literature search strategy used for the identification of articles on ataxin-2 as a risk factor for ALS.**
(DOCX)
