# Supplementary material for: Intermediate CAG Repeat Expansion in the ATXN2 Gene Is a Unique Genetic Risk Factor for ALS−A Systematic Review and Meta-Analysis of Observational Studies
Source: PLoS One. 2014 Aug 22;9(8):e105534. doi: 10.1371/journal.pone.0105534 (PMC4141758; doi:10.1371/journal.pone.0105534)
Supplement: Table S1 — Summary of studies included in meta-analyses of the ATXN2 gene as a risk factor for ALS. (DOCX) [file pone.0105534.s001.docx]

Table S1. Summary of studies included in meta-analyses of the ATXN2 gene as a risk factor for ALS

| **Author, Year, Country** | **ALS or MND Diagnosis** | **Sporadic, Familial sex, Case, Control and polyQ information** | **Main results and Conclusion** | Ref No. |
| --- | --- | --- | --- | --- |
| Included studies | |  |  |  |
| Conforti, 2012, Italy | ALS, EI Escorial, exclude SOD1, TDP-43, ANG, FUS, C9ORF72 positive, Control matched with geographic | 1. 405 sALS:  2. 13 fALS.  3. 296 control: | 1. 57 out of 806 *ATXN1* alleles in sALS cohort harbored a >=32 polyQ repeat (7.07%), compared to 13 out of 544 NC alleles (2.38%, p=0.0001). OR=2.396(1.26-4.56)  2. For *ATXN2*, 22 X >=28Q in 808 sALS alleles (2.72%) and only 3 (0.5%) of 586 NC alleles (p=0.01). OR=5.832(1.71-9.78). | (1) |
| Corrado, 2011, Italy | ALS, EI Escorial. Screened SOD1, TDP-43, FUS, regionally matched controls | 1. 232 ALS (219 sALS, 13 fALS ): 1X24Q; 1X27Q, 0X28Q; 1X29Q; 0X30Q; 3X31Q; 1X32Q; 2X33Q; 1X37Q.  2. 395 controls: 3X24Q; 6X27Q, 1X28Q; 4X29Q; 1X30Q; 0X31Q; 0X32Q; 0X33Q; 0X37Q. | 1. Significantly association.  2. No observation for symptoms of ataxia, dementia or other atypical features. | (2) |
| Daoud H, 2011, France/Qubec, Canada | 1. ALS, EI Escorial, probable, or definite cases, diagnosed by ALS specialists. FALS were sod1, tardbp, fus, vapb, ang free. Neurological health controls matched by age, ethnicity. Not mentioned sex, and recruitment time. | 1. 461 sALS: >=32, 9;  2. 95 fALS: >=32, 2;  3. 471 unrelated controls. Range from 19-32. 19-37 in patients. 24 of 471 healthy controls (5,1%) harboured 1 intermediate *ATXN2* allele(range 24-33), whereas 40 of 556 cases (7.2%) had one allele in that range (p=0.15).  4. Figure may help do scientific guess. | 1, ROC showed that 27 cut-off gave best sensitivity/specificity. 19 from controls (4,1%), compared to 35 from cases(6.3%) within this range (>=27), no difference(p=0.09). The p becomes significant when compared within the range of >=29. 4 controls (0.8%), and 25 cases (18/461, 4.5%). OR=5.5(1.9-15.9). 7 out of 95 fALS (7.4%), 18 out of 461 sALS cases: OR for FALS was 9.29(2.66-32.4); for sALS is 4.74(1.59-14.13). When >=32 remove, the difference is still significant (14 from cases, 3 from controls, OR=4.03(1.15-14.11)) for the range between 29-31.  2. Length is not associated with age at onset (>=29) in cases were no >=29 cases. | (3) |
| Elden, 2010, USA | ALS, recruited in PA and NJ, no recruitment time, no case control design | 1. 915 ALS. 6X24Q; 1X25Q; 0X26Q; 22X27Q; 1X28Q; 2X29Q; 4X30Q; 7X31Q; 8X32Q; 2X33Q.  2. 980 Controls: 4X24Q; 4X25Q; 2X26Q; 11X27Q; 0X28Q; 1X29Q; 0X30Q; 2X31Q; 0X32Q; 0X33Q | 1. CAG intermediate repeat expansion is associated with ALS.  2. The repeat might be a modulator. | (4) |
| Gellera, 2012, Italy | ALS, EI Escorial, SOD1 free, for all, FALS also screened ANG, TDP-43, FUS, C9ORF72 (carriers of FALS cases-separated) | 1. 658 SALS: 4 X 24-26Q; 10X27Q, 4X 29Q; 3X30Q; 7X31Q; 6X32Q; 2X33Q. No >33Q.  2. 41 FALS-G(carriers): 1 X 27-29Q; 1X30Q. No others  3. 102 FALS-Unknown: 2 X 24-26Q; 1X 27-29Q; 1X30Q.  4.231 Sporadic ataxic patients: 5X 24-26Q; 6X27-29Q; 2X30Q. No others.  4. 551 Health controls: 6X24-26Q; 18X27-29Q; 1X30Q; 1X31Q. No others. | 1. The frequency of ATXN2 alleles with 27-30 repeats was similar in SALS and control subjects.  2. Fifteen SALS subjects carried ≥ 31 CAG repeats. This difference was statistically significant (p = 0.0014). No alleles with ≥ 34 CAG were found.  3. In FALS, the distribution of ATXN2 alleles was similar to control subjects. | (5) |
| Gispert, 2012, Germany | ALS, European ALS clinics (diagnostic criteria), | 1. 559 ALS (included 89 fALS, 1X 32Q): 1X24Q, 1X25Q; 0X26Q; 15X27Q; 0X28Q; 1X29Q; 3X30Q; 0X31Q; 3X32Q; 0X33Q; 0X34Q; 1X35Q.  2. 1378 controls: 9X24Q, 1X25Q; 0X26Q; 48X27Q; 1X28Q; 5X29Q; 1X30Q; 1X31Q; 0X32Q; 0X33Q; 1X34Q; 0X35Q.  3. 1142 PD: 6X24Q, 2X25Q; 1X26Q; 37X27Q; 1X28Q; 5X29Q; 0X30Q; 2X31Q; 1X32Q; 0X33Q; 0X34Q; 3X37Q; 1X39Q; 1X40Q. | 1. In 559 sporadic ALS patients from Central Europe, the association of ATXN2 expansions (30≤polyQ≤35) with ALS was highly significant.  2. The study of 1490 patients with Parkinson's disease (PD) showed an enrichment of ATXN2 alleles 27/28 in a subgroup with familial cases, but the overall risk of sporadic PD was unchanged.  3. No association was found between polyQ expansions in Ataxin-3 (ATXN3) and ALS risk. | (6) |
| Lahut, 2012, Turkey | ALS, no other information | 1. 236 ALS. 4X24Q; 1X25Q; 3X27Q; 1X28Q; 1X29Q; 1X31Q; 3X32Q.  2. 420 Healy controls. 3X24Q; 0X25Q; 1X27Q; 0X28Q; 0X29Q; 0X31Q; 0X32Q. | 1. 15 ALS patients carrying SOD1, UBQLN2, OPTN, SPG11, or PLEKHG3 were intermediate repeat negative.  2. 4 X >31Q (1fALS, 3sALS). Could not find any information about total FALS cases.  2. Calculate >30 in this study. 3 out of 4 patients with 31 and 32Q had a single CAA interruption. Not in other ALS cases. | (7) |
| Lee T, 2011, Multiple European countries | ALS, EI Escorial, Controls matched by age and gender, who were either the spouses of ALS patients, healthy donors. Did not mentioned recruitment time | 1. 400 fALS: >30, 6;  2. 894 sALS: >30. 7;  3.679 controls. 20 (2.9%) out of 679 controls harboured intermediate polyQ (range 27-30). 45 out of 1294 als patients (3.5%, range 27-35). For >30 repeat, no in 679 controls, but found 14 cases among 1294 ALS patients (p=0.0062). | 1. No ataxia, dementia was observed in ALS patients.  2. No difference compared with and without repeat for age at onset, disease duration,.  3. Intermediate-length ataxin 2 polyQ repeat expansions are associated with increased risk for ALS also in the European cohort. The specific polyQ length cut-off, however, appears to vary between different populations, with longer repeat lengths showing a clear association. | (8) |
| Ross, USA, Canada, 2011 | 1. EI Escorial. Recruited from 2008-2010 | 1. 532 als: >=27Q, 33(6.2%); >=31Q, 8(1.5%).  2. 4877 control: >=27Q, 197(4.0%); >=31Q, 9(0.2%).  3. 642 FTD: >=27Q, 31(4.8%); >=31Q, 9(0.5%).  4. 1530 AD: >=27Q, 56(3.7%); >=31Q, 3(0.2%).  5. 514 PSP: >=27Q, 24(4.7%); >=31Q, 4(0.8%).  6.702 PD: >=27Q, 28(4.0%); >=31Q, 2(0.3%). | 1. ALS, for >=27, OR=1.58(1.08-2.31); for >=31, OR=5.57(1.95-15.88).  2. FTD, for >=27, OR=1.20(0.82-1.76); for >=31Q, OR=1.94(0.51-7.37)  3. for AD, for >=27, OR=0.96(0.70-1.33); for >=31Q, OR=2.17(0.40-11.96)  4. For PSP, for >=27, OR=1.20(0.78-1.85); for >=31Q, OR=5.83(1.74-19.52)  5. For PD, for >=27, OR=0.95(0.63-1.43); for >=31Q, OR=0.93(0.19-4.51).  Author speculated that long Q repeat in controls (9 controls) might be due to young age (reduced disease penetrance). SCA2 not CAA interruption? | (9) |
| Soraru, 2011, Italy | ALS, EI Escorial, recruited from 01 of 2004 to 08 of 2010. Did not mention control match | 1. 247 ALS. >=24Q, 17(6.8%). 3 X24Q, 1X 26Q, 6X 27Q, 2X30Q, 1X31Q, 4X32Q  2. 256 controls: >=24Q, 6(2.3%). 1X24Q, 2X27Q, 1X28Q, 2X31Q. | 1. Intermediate polyQ is more frequent in ALS patients, than in controls (p=0.026).  2. No difference was observed for age at onset, bulbar/spinal onset ratio, survival time, etc. | (10) |
| Van Damme, 2011, Belgium/Netherlands | 1. ALS, EI Escorial. 1995-2010, neurological conditions free normal controls | 1. 1845 SALS. >=32, 10(5 X 32, 2X33, 1X34, 1X36, 1X39; 0.5%); (31,4; 30, 5; 29,9; 28, 1; Scientific guess from fig. we can further guess 27 repeat)  2. 103 fALS cases from 91 families. but sod1, fus, TARDBP, ANG free. 2/91 (2.2%) long repeat, 1/91, 31 repeat; 1/91, 33 repeat;  3. 2002 controls. Range, 16-31; 22, 90.1%; 23, 6.1%; 27, 1.7%; 31, 0.1% (heterozygous, 0.2%). (31,5; 30, 4; 29,7; 28, 1; Scientific guess from fig.) | 1. p=0.0006 for repeat >=32 between ALS and control. No difference for <=31 (22-31, or 27-31 or 29-31).  2. ROC curve show a cutoff >=29 yield greatest sn, spn.28 out of ALS patients (1.5%) versus 16 out of 2002 controls (p=0.036, OR=1.92(1.04-3.64)). Combined with an American study (915 ALS, 980 controls), OR=2.93(1.73-4.98).  3. No association with survival, age at onset, site of onset.  4. Pedigree (33:33), onset at 71, and his 2 y elder als brother (31:33, onset at 75), normal brother (22:33) were described from consanguineous family. One of their parents was possible affected by ALS. No ataxia or cerebellar degeneration was found. | (11) |
| Studies from China | |  |  |  |
| Chen, 2011, China | 1. ALS, EI Escorial, 05-2004-06-2010. Excluded fALS, Community controls matched by age, sex, race from same period. | 1. 345 sALS (254 spinal, 91 bulbar). >=24, 15; <24, 330; >=27, 12; <27, 333; >=28, 11; >=29, 8; >=31, 4.  2. 350 controls (17-30). >=24, 8; <24, 342; >=27, 4; <27, 346; >=28, 3; >=29, 2; >=31, 0.  3. Provide a table for comparing repeat length and clinic features | 1. Mean age of onset, gender, and onset site between with and without *ATXN2*, no difference.  2. p=0.040 for >=27.  3. Mean age of onset (for >=31), is longer than (<31): 44.5±8.5y verus51.57±12.51, p-0.197 | (12) |
| Liu, 2013, China | ALS, EI Escorial, whole China, did not state the recruitment time, no control match information | 1. 1067 als: 4X26Q; 4X 27Q; 3X28Q; 6X29Q; 3X30Q; 17 X >30Q (6X31Q; 5X32Q; 3X33Q; 2X34Q; 1X35Q).  2. 506 healthy: 1X26Q; 2X 27Q; 4X28Q; 0X29Q; 7X30Q; 0X >30Q.  3> 6 fALS, no. | 1. Association of ALS with ataxin-2 intermediate CAG repeats was confirmed.  2. No clinical manifestation associated with repeat observed. | (13) |
| Excluded article | |  |  |  |
| Van Langenhove, 2012, Belgium | No diagnostic, recruitment and control information. | 1. 72 ALS cases, including 18 fALS. 27-33Q, 7; 30-33Q, 3; >31Q, 1 (33Q). No information for fALS alone.  2. 22 FTLD-ALS: 27-33Q, 1; >31Q, 0.  3. 270 FTLD. 27-33Q, 8;  4. 810 controls.27-33Q, 25; >31, 0. | 1. Significant phenotype overlap between ALS and SCA2 was observed.  2. Intermediate repeat of polyQ is associated with fALS.  3. No similar association was identified for FTLS and FTLD-ALS. | (14) |
| Multiple use of same sample | | Title | Main results |  |
| Bonini, 2011, USA | Same as Elden | Model organisms reveal insight into human neurodegenerative disease: ataxin-2 intermediate-length polyglutamine expansions are a risk factor for ALS | 1. For 27-33Q; 1.4% in control; 4.7% in ALS (including sALS and fALS). Significantly associated intermediate polyQ repeats with ALS.  2. Mentioned that the cut-off appeared dependent on the specific population.  3. This study used similar approaches as in Elden’s paper | (15) |
| Lee T, 2011, USA | Same as Elden | Evaluating the prevalence of polyglutamine repeat expansions in amyotrophic lateral sclerosis | 1. Assessed the polyQ lengths of ataxin 1, ataxin 3, ataxin 6, ataxin 7, TBP, atrophin 1,and huntingtin in several hundred patients with sporadic ALS and healthy controls.  2. Other than ataxin 2, we did not identify a significant association with the other polyQ genes and ALS | (16) |
| Yu Z,  2011, USA | Same as Elden | PolyQ repeat expansions in ATXN2 associated with ALS are CAA interrupted repeats | Expanded repeat alleles of 40 ALS patients and 9 long-repeat length controls were all interrupted, bearing 1–3 CAA codons within the CAG repeat. | (17) |

**Bibliography**

(1) Conforti FL, Spataro R, Sproviero W, Mazzei R, Cavalcanti F, Condino F, et al. Ataxin-1 and ataxin-2 intermediate-length PolyQ expansions in amyotrophic lateral sclerosis. Neurology Dec 2012;79(24):2315-2320.

(2) Corrado L, Mazzini L, Oggioni GD, Luciano B, Godi M, Brusco A, et al. ATXN-2 CAG repeat expansions are interrupted in ALS patients. Hum Genet 2011 October 2011;130(4):575-580.

(3) Daoud H, Belzil V, Martins S, Sabbagh M, Provencher P, Lacomblez L, et al. Association of long ATXN2 CAG repeat sizes with increased risk of amyotrophic lateral sclerosis. Arch Neurol Jun 2011;68(6):739-742.

(4) Elden AC, Kim H-, Hart MP, Chen-Plotkin AS, Johnson BS, Fang X, et al. Ataxin-2 intermediate-length polyglutamine expansions are associated with increased risk for ALS. Nature 2010 26 Aug 2010;466(7310):1069-1075.

(5) Gellera C, Ticozzi N, Pensato V, Nanetti L, Castucci A, Castellotti B, et al. ATAXIN2 CAG-repeat length in Italian patients with amyotrophic lateral sclerosis: Risk factor or variant phenotype? Implication for genetic testing and counseling. Neurobiol Aging Aug 2012;33(8):e15-e21.

(6) Gispert S, Kurz A, Waibel S, Bauer P, Liepelt I, Geisen C, et al. The modulation of Amyotrophic Lateral Sclerosis risk by ataxin-2 intermediate polyglutamine expansions is a specific effect. Neurobiol Dis 2012 Jan;45(1):356-361.

(7) Lahut S, Omur O, Uyan O, Agim ZS, Ozoguz A, Parman Y, et al. ATXN2 and its neighbouring gene SH2B3 are associated with increased ALS risk in the Turkish population. PLoS One 2012;7(8):e42956.

(8) Lee T, Li YR, Ingre C, Weber M, Grehl T, Gredal O, et al. Ataxin-2 intermediate-length polyglutamine expansions in European ALS patients. Hum Mol Genet 2011;20(9):1697-1700.

(9) Ross OA, Rutherford NJ, Baker M, Soto-Ortolaza AI, Carrasquillo MM, DeJesus-Hernandez M, et al. Ataxin-2 repeat-length variation and neurodegeneration. Hum Mol Genet 2011 Aug 15;20(16):3207-3212.

(10) Soraru G, Clementi , M, Forzan , M, Orsetti V, D'Ascenzo C, Querin G, et al. ALS risk but not phenotype is affected by ataxin-2 intermediate length polyglutamine expansion. Neurology Jun 2011;76(23):2030-2031.

(11) Van Damme P, Veldink JH, van Blitterswijk M, Corveleyn A, van Vught, P. W. J, Thijs V, et al. Expanded ATXN2 CAG repeat size in ALS identifies genetic overlap between ALS and SCA2. Neurology Jun 2011;76(24):2066-2072.

(12) Chen Y, Huang R, Yang Y, Chen K, Song W, Pan P, et al. Ataxin-2 intermediate-length polyglutamine: A possible risk factor for Chinese patients with amyotrophic lateral sclerosis. Neurobiol Aging Oct 2011;32(10):e1-e5.

(13) Liu X, Lu M, Tang L, Zhang N, Chui D, Fan D. ATXN2 CAG repeat expansions increase the risk for Chinese patients with amyotrophic lateral sclerosis. Neurobiol Aging Sep 2013;34(9):e5-e8.

(14) Van Langenhove T, van der Zee J, Engelborghs S, Vandenberghe R, Santens P, Van den Broeck M, et al. Ataxin-2 polyQ expansions in FTLD-ALS spectrum disorders in Flanders-Belgian cohorts. Neurobiol Aging May 2012;33(5):e17-e20.

(15) Bonini NM, Gitler AD. Model organisms reveal insight into human neurodegenerative disease: ataxin-2 intermediate-length polyglutamine expansions are a risk factor for ALS. J Mol Neurosci 2011 Nov;45(3):676-683.

(16) Lee T, Li YR, Chesi A, Hart MP, Ramos D, Jethava N, et al. Evaluating the prevalence of polyglutamine repeat expansions in amyotrophic lateral sclerosis. Neurology Jun 2011;76(24):2062-2065.

(17) Yu Z, Zhu Y, Chen-Plotkin AS, Clay-Falcone D, McCluskey L, Elman L, et al. PolyQ repeat expansions in ATXN2 associated with ALS are CAA interrupted repeats. PLoS One 2011 Mar 29;6(3):e17951.
