# Supplementary material for: Intermediate CAG Repeat Expansion in the ATXN2 Gene Is a Unique Genetic Risk Factor for ALS−A Systematic Review and Meta-Analysis of Observational Studies
Source: PLoS One. 2014 Aug 22;9(8):e105534. doi: 10.1371/journal.pone.0105534 (PMC4141758; doi:10.1371/journal.pone.0105534)
Supplement: Table S2 — Age at onset, disease duration and CAG repeat number in ALS patients. (DOCX) [file pone.0105534.s002.docx]

Table S2: Age at onset, disease duration and CAG repeat number in ALS patients

| Reference | Patient ID | Sex | Age at onset (years) | Disease duration ( years) | CAG Repeat | ALS FORM | ALS TYPE |
| --- | --- | --- | --- | --- | --- | --- | --- |
| Chen, 2011,China | A1 | F | 34 | 4.1 | 30 |  |  |
|  | A2 | F | 32 | 3.3 | 27 |  |  |
|  | A3 | F | 48 | 2.1 | 25 |  |  |
|  | A4 | M | 40 | 1.9 | 28 |  |  |
|  | A5 | M | 45 | 1.4 | 31 |  |  |
|  | A6 | F | 56 | 5.6 | 29 |  |  |
|  | A7 | M | 41 | 4.5 | 31 |  |  |
|  | A8 | M | 36 | 1.4 | 31 |  |  |
|  | A9 | M | 37 | 3.9 | 28 |  |  |
|  | A10 | M | 45 | 4.4 | 25 |  |  |
|  | A11 | M | 56 | 4.2 | 31 |  |  |
|  | A12 | F | 64 | 4.2 | 24 |  |  |
|  | A13 | M | 55 | 2.6 | 30 |  |  |
|  | A14 | F | 57 | 5.3 | 28 |  |  |
|  | A15 | M | 45 | 4.2 | 30 |  |  |
| Daoud, 2011,Canada | 1 | F | MISSING |  | 32 | Familial |  |
|  | 2 | M | 75 |  | 32 | Familial |  |
|  | 3 | M | 75 |  | 32 | Sporadic |  |
|  | 4 | F | 76 |  | 32 | Sporadic |  |
|  | 5 | M | 67 |  | 32 | Sporadic |  |
|  | 6 | M | 58 |  | 37 | Sporadic |  |
|  | 7 | M | 60 |  | 32 | Sporadic |  |
|  | 8 | M | 52 |  | 35 | Sporadic |  |
|  | 9 | F | 76 |  | 35 | Sporadic |  |
|  | 10 | M | 72 |  | 36 | Sporadic |  |
|  | 11 | F | 76 |  | 32 | Sporadic |  |
| Corrado, 2011, Italy | 2294 | F | 63 | 13 | 31 | ALS-LMN | LUMBER |
|  | 2309 | M | 66 | 2.2 | 31 | ALS | CERVICAL |
|  | 4464 | M | 69 | 1.8 | 31 | ALS | LUMBER |
|  | 5421 | M | 74 | 2.5 | 32 | ALS | CERVICAL |
|  | 5304 | M | 44 | 1.8 | 33 | ALS | BULBAR |
|  | 2282 | M | 58 | 7 | 33 | ALS | CERVICAL |
|  | 3063 | M | 60 | 0.3 | 37 | ALS | LUMBER |
| van Damme, 2011,Belgium | 1 | M | 52 | 0.8 | 32 |  | SPINAL |
|  | 2 | F | 56 | 3.1 | 32 |  | SPINAL |
|  | 3 | F | 42 | 2.9 | 32 |  | SPINAL |
|  | 4 | M | 62 | 2.1 | 32 |  | SPINAL |
|  | 5 | F | 69 | ALIVE(38M) | 32 |  | SPINAL |
|  | 6 | F | 54 | ALIVE(29M) | 33 |  | SPINAL |
|  | 7 | M | 35 | ALIVE(13M) | 33 |  | SPINAL |
|  | 8 | F | 52 | 3.8 | 34 |  | SPINAL |
|  | 9 | F | 80 | 4.9 | 36 |  | BULBAR |
|  | 10 | M | 71 | ALIVE(15) | 39 |  | SPINAL |
| Ross, 2011, USA | 1 | F | 54 | 1 | 36 | ALS |  |
|  | 2 | M | 78 | 1 | 32 | ALS |  |
|  | 3 | M | 54 | N/A | 32 | ALS |  |
|  | 4 | M | 66 | N/A | 32 | PMA |  |
|  | 5 | F | 79 | 2 | 32 | ALS |  |
|  | 6 | F | 58 | 3 | 31 | ALS |  |
|  | 7 | M | 58 | N/A | 31 | ALS |  |
| Yu, 2011, USA | A32 |  | 49 |  | 29 |  |  |
|  | A48 |  | 72 |  | 29 |  |  |
|  | A35 |  | 62 |  | 30 |  |  |
|  | A33 |  | 72 |  | 30 |  |  |
|  | A42 |  | 74 |  | 31 |  |  |
|  | A37 |  | 61 |  | 31 |  |  |
|  | A43 |  | 58 |  | 31 |  |  |
|  | A50 |  | 77 |  | 32 |  |  |
|  | A47 |  | 79 |  | 32 |  |  |
|  | A51 |  | 55 |  | 32 |  |  |
|  | A49 |  | 53 |  | 32 |  |  |
|  | A53 |  | 52 |  | 33 |  |  |
|  | A52 |  | 70 |  | 33 |  |  |
|  | A30 |  | 55 |  | 28 |  |  |
|  | A41 |  | 35 |  | 29 |  |  |
|  | A34 |  | 58 |  | 30 |  |  |
|  | A38 |  | 56 |  | 31 |  |  |
|  | A40 |  | 65 |  | 31 |  |  |
|  | A45 |  | 54 |  | 32 |  |  |
|  | A14 |  | 30 |  | 27 |  |  |
|  | A17 |  | 65 |  | 27 |  |  |
|  | A16 |  | NOT KNOWN |  | 27 |  |  |
|  | A18 |  | 53 |  | 27 |  |  |
|  | A19 |  | 59 |  | 27 |  |  |
|  | A20 |  | 57 |  | 27 |  |  |
|  | A21 |  | 60 |  | 27 |  |  |
|  | A22 |  | 46 |  | 27 |  |  |
|  | A23 |  | 49 |  | 27 |  |  |
|  | A24 |  | 53 |  | 27 |  |  |
|  | A12 |  | 66 |  | 27 |  |  |
|  | A25 |  | 48 |  | 27 |  |  |
|  | A27 |  | 51 |  | 27 |  |  |
|  | A26 |  | 47 |  | 27 |  |  |
|  | A28 |  | 62 |  | 27 |  |  |
|  | A10 |  | 51 |  | 27 |  |  |
|  | A11 |  | 61 |  | 27 |  |  |
|  | A13 |  | 59 |  | 27 |  |  |
|  | A31 |  | 64 |  | 29 |  |  |
|  | A39 |  | 63 |  | 31 |  |  |
|  | A46 |  | 42 |  | 32 |  |  |
| Hart, 2012, USA | 1 | M | 41 | 1 | 24 | FALS |  |
|  | 2 | F | 47 | 3 | 22 | ALS |  |
|  | 3 | M | 75 | 1 | 22 | ALS |  |
|  | 4 | M | 56 | 18 | 22 | ALS |  |
|  | 5 | F | 79 | 2 | 22 | ALS |  |
|  | 6 | M | 41 | 3 | 22 | ALS |  |
|  | 7 | M | 45 | 9 | 22 | ALS |  |
|  | 8 | F | 55 | N/A | 22 | ALS |  |
|  | 9 | M | 59 | 3 | 22 | YES |  |
|  | 10 | F | 50 | 1 | 22 | ALS |  |
|  | 11 | F | 48 | 3 | 22 | ALS |  |
|  | 12 | F | 73 | 1 | 22 | ALS |  |
|  | 13 | M | 39 | N/A | 22 | ALS |  |
|  | 14 | F | 54 | 2 | 27 | ALS |  |
|  | 15 | F | 30 | 13 | 27 | YES |  |
|  | 16 | M | 78 | 0 | 27 | ALS |  |
|  | 17 | F | 65 | 3 | 31 | ALS |  |
|  | 18 | F | N/A | N/A | 32 | ALS |  |
|  | 19 | M | N/A | N/A | 32 | ALS |  |
| Gispert,2012, Germany | 1 | M | 41 | N/A | 35 |  |  |
|  | 2 | F | 66 | 2 | 30 |  |  |
|  | 3 | M | 72 | N/A | 30 |  |  |
|  | 4 | F | 66 | 1 | 32 |  |  |
|  | 5 | M | 71 | 2 | 30 |  |  |
|  | 6 | M | 65 | 9 | 32 |  |  |
|  | 7 | F | 72 | 3 | 32 |  |  |
| Lahut, 2012,Turkey | 1 | F | 52 | 3 | 31 | ALS |  |
|  | 2 | M | 39 | 5 | 32 | ALS |  |
|  | 3 | F | 77 | LIVE | 32 | ALS |  |
|  | 4 | F | 8 | LIVE | 32 | FALS |  |
