# Supplementary material for: Intermediate CAG Repeat Expansion in the ATXN2 Gene Is a Unique Genetic Risk Factor for ALS−A Systematic Review and Meta-Analysis of Observational Studies
Source: PLoS One. 2014 Aug 22;9(8):e105534. doi: 10.1371/journal.pone.0105534 (PMC4141758; doi:10.1371/journal.pone.0105534)
Supplement: Search S1 — Literature search strategy used for the identification of articles on ataxin-2 as a risk factor for ALS. (DOCX) [file pone.0105534.s004.docx]

Search S1. Literature search strategy used for the identification of articles on ataxin-2 as a risk factor for ALS

The search strategy employed to identify articles relevant to our investigation of the ataxin-2 gene as a risk factor for amyotrophic lateral sclerosis (ALS) included three separate searches of different bibliographic databases, each with unique search criteria designed specifically for those databases.

**A. Medline, Psycinfo, and Embase**

The search of these three databases applied following 21 search steps.

1. amyotrophic lateral sclerosis

2. motor neuron disease

3. motoneurone disease

4. Lou Gehrig's disease

5. als

6. mnd

7. 1 or 2 or 3 or 4 or 5 or 6

8. ataxin-2

9. *ATXN2*

10. atx2

11. sca2

12. asl13

13. tnrc13

14. polyglutamine

15. polyq

16. cag repeat

17. 8 or 9 or 10 or 11 or 12 or 13 or 14 or 15 or 16

18. 7 and 17

19. limit 18 to english language

20. limit 19 to human

21. limit 20 to yr="2010 -Current"

A total of 216 potentially relevant articles were identified through this search.

**B. Hugenet Navigator**

1. First select ‘literature’ option, then enter disease term: amyotrophic lateral sclerosis

2. Filter with gene: *ATXN2*

A total of 11 potentially relevant articles were identified through this search.

**C. Pubmed**

(ALS, MND, amyotrophic lateral sclerosis, motor neuron disease, motorneurone disease, or Lou Gehrig’s disease) and (ataxin-2, *ATXN2*, *ATX2*, *SCA2, ASL13, TNRC13*, polyglutamine, or polyQ).

A total of 45 articles were identified through this search. .
